# Cross-organ protection of MSC-derived extracellular vesicles in ischemia-reperfusion injury: angiogenic synergy in kidney, brain, and heart

**DOI:** 10.3389/fcvm.2025.1634877

**Published:** 2025-08-19

**Authors:** Zhuhong Lai, Dong Li, Caidong Luo, Qingyan Qiu, Rong Li, Min Dai

**Affiliations:** Department of Cardiology, Mianyang Central Hospital, School of Medicine, University of Electronic Science and Technology of China, Mianyang, China

**Keywords:** kidney, brain, heart, mesenchymal stem cell, extracellular vesicles, angiogenic, ischemia-reperfusion injury

## Abstract

Ischemia-reperfusion injury, marked by transient blood flow disruption followed by tissue reperfusion, constitutes a unifying pathological mechanism across cerebral stroke, myocardial infarction, and acute kidney injury. Hypoxia, a central driver of ischemia-reperfusion injury progression, triggers molecular cascades that simultaneously exacerbate tissue damage and activate compensatory repair mechanisms. Notably, hypoxia-induced angiogenesis and vascular remodeling serve as critical adaptive processes for functional recovery, supporting neuronal plasticity in stroke, myocardial salvage in infarction, and tubular regeneration in renal ischemia-reperfusion injury. While these conditions exhibit organ-specific manifestations, emerging studies underscore conserved regulatory frameworks mediated by extracellular vesicles (EVs) and their molecular cargoes, which orchestrate cross-organ protective responses. In this context, mesenchymal stem cell (MSC)-derived EVs have emerged as potent therapeutic agents for mitigating ischemia-reperfusion injury-related deficits, as evidenced by preclinical and clinical studies. These EVs act as bioactive nanocarriers, delivering cargos that modulate shared pathological pathways-particularly angiogenesis, a linchpin of post-ischemic tissue repair. Accumulating evidence highlights cargos within MSC-EVs (e.g., miRNAs, proteins) as master regulators of vascular regeneration, fine-tuning endothelial proliferation, vessel maturation, and hypoxia adaptation. This review systematically examines the dual roles of MSC-EV-associated cargos in promoting or suppressing angiogenesis across cerebral, cardiac, and renal ischemia-reperfusion injury models. By dissecting their mechanisms in spatiotemporal regulation of vascular signaling networks, we aim to elucidate their translational potential as universal therapeutic targets for multi-organ ischemia-reperfusion injury management.

## 1 Introduction

Ischemia-reperfusion injury is a critical pathological process characterized by oxidative stress, inflammation, and endothelial dysfunction, commonly affecting highly vascularized organs such as the kidney, brain, and heart (1). Recent studies highlight that angiogenic synergy plays a pivotal role in lessening ischemia-reperfusion injury-induced tissue damage and promoting vascular repair across these organs (2). These conditions share underlying disease pathways, including overlapping cytokine signatures, inflammatory agents, and especially angiogenesis-associated elements, which will be discussed in detail. A large number of these angiogenic components are secreted through autocrine or paracrine signaling pathways, primarily via EVs, allowing them to circulate systemically and influence distant tissues, including the central nervous system (3–5). Notably, EVs can cross the blood-brain barrier (BBB) to deliver therapeutic agents directly to ischemic brain tissue, a capability rare in other treatments (6).

EVs serve as vital mediators of long-range cellular communication in multicellular organisms, offering protection to their cargo from enzymatic breakdown by enclosing it within a lipid bilayer, which restricts enzyme access (7, 8). This unique property of EVs facilitates the phenomenon of angiogenic synergy across the kidney, brain, and heart. A particular subset of EVs are secreted by mesenchymal stem cells (MSCs) and are involved in intercellular signaling. These MSC-EVs exhibit immunoregulatory, anti-inflammatory, and tissue-repairing properties across a range of disease states (9). Evidence indicates that MSC-EVs actively mitigate ischemic injury across multiple organs, including the kidney, brain, and hear (10–12). Furthermore, MSC-EVs not only express surface markers typical of MSCs but also transport a diverse array of angiogenesis-related molecules (11, 13). At the molecular level, MSC-EVs deliver pro-angiogenic miRNAs (e.g., miR-126, miR-132), growth factors (such as VEGF, FGF2), and signaling molecules that activate pathways like PI3 K/Akt and MAPK/ERK, which are vital for endothelial cell proliferation and migration (14).

This study interrogates the multi-organ defense conferred by mesenchymal-stem-cell-derived extracellular vesicles (MSC-EVs) against ischemia–reperfusion injury, centering on their capacity to orchestrate a tri-organ angiogenic alliance among kidney, brain, and heart. By elucidating how MSC-EVs synchronize pan-vascular repair and calibrate systemic immunity, this studt aims to advance these vesicles as a next-generation, cell-free therapy for integrated multi-organ ischemia–reperfusion injury.

## 2 MSC-derived extracellular vesicles

EVs are membrane-bound particles encased within a lipid bilayer, secreted by virtually all cell types under both physiological and pathological conditions (15). They are essential mediators of complex, long-range communication between cells in multicellular systems, as their lipid bilayer safeguards internal cargo from enzymatic breakdown by blocking enzyme access (7, 8). The role of EVs in the development and progression of numerous ischemic disorders has become increasingly evident, largely due to the functional variety of the biomolecules they carry (16–19). Under pathological conditions, these EV-encapsulated cargos contribute to angiogenic activity across a range of ischemic conditions, including myocardial ischemia (20), renal ischemia (21), and ischemic stroke (22), which we will illustrate in details.

EVs are commonly grouped into three categories according to their size and formation process: exosomes (typically under 150 nm in diameter), microvesicles (MVs) (ranging between 100 and 1,000 nm), and apoptotic bodies (with diameters between 0.8 and 5.0 µm) (23). Nevertheless, due to the difficulty in definitively tracing an EV to its exact origin pathway, the International Society for Extracellular Vesicles (ISEV) advises using descriptive terms based on measurable physical features. For instance, the term “small EVs” (sEVs) generally refers to particles smaller than 100–200 nm, whereas “medium/large EVs” (m/l EVs) describes those exceeding 200 nm in size. EVs may also be classified based on their density into low-, medium-, or high-density groups, with each category having defined criteria (24). EVs are secreted by all cell types and can be found in nearly all body fluids, including blood (25), saliva (26), cerebrospinal fluid (CSF) (27), breast milk (28), urine (29), and semen (30), underscoring their promising value of EVs in clinical biomarker studies. This widely spread of EVs in both intra- and extrathecal make the Angiogenic Synergy function between ischemia disease matters.

MSCs are multipotent cells capable of self-renewal and differentiating into various cell types when introduced into suitable tissue environments (31). They can be sourced from various tissues in both adults and neonates. MSCs are known for their unique immunomodulatory properties, which allow them to evade immune recognition and suppress immune responses (32). As an emerging therapeutic tool, MSCs have attracted considerable interest due to their strong proliferative potential, minimal immunogenicity, capacity for differentiation into multiple lineages, and relatively minor ethical concerns compared to other stem cell sources (31, 33).

MSC-EVs are nanoscale, membrane-enclosed particles released by MSCs that facilitate cell-to-cell signaling and contribute to immune regulation, inflammation reduction, and tissue repair under various pathological conditions (9). In addition, MSC-EVs have been shown to play a vital role in treating ischemic injuries across multiple organs, such as the kidney, brain, and heart (10, 34, 35). These vesicles not only present markers typical of MSCs-such as CD29, CD73, CD90, CD44, and CD105-but also contain numerous angiogenesis-related molecules, including miR-543 and miR-210 (11, 36). Consequently, MSCs sourced from various tissues have been explored for therapeutic use in managing ischemia-related damage (37–40).

## 3 The effect of MSC-EVS on angiogenesis following myocardial ischemia- reperfusion injury

Myocardial ischemic injury occurs when reduced or interrupted blood flow in the coronary arteries leads to damage and necrosis of the myocardium due to ischemia and hypoxia (41). Common causes include coronary atherosclerosis, thrombus formation, or coronary artery spasm (41, 42). Ischemia-reperfusion injury of the myocardium, in contrast, refers to the exacerbation of myocardial cell damage and death due to the production of harmful substances (such as reactive oxygen species and reactive nitrogen species) when blood flow is restored after myocardial ischemia (42). Myocardial infarction, the most prevalent form of myocardial ischemic injury, is also one of the leading causes of death and disability worldwide (43). When the heart encounters blood supply impairment, improving cardiac blood supply, promoting the regeneration of new blood vessels and the repair of damaged vessels, as well as reducing myocardial injury, fibrosis, and enhancing myocardial function, have become crucial directions for improving and treating myocardial ischemic diseases and ischemia-reperfusion injuries (43, 44). In recent years, MSC-EVs have emerged as a research hotspot due to their unique biological properties and therapeutic potential. Studies have shown that MSCs from various sources can secrete EVs that act on damaged tissue cells to promote angiogenesis, increase vascular density, reduce myocardial fibrosis, and decrease myocardial apoptosis, thereby alleviating myocardial ischemic injury and reperfusion injury (20, 45). To gain a comprehensive understanding of the effects of MSC-EVs on myocardial ischemic diseases, researchers have conducted literature searches on websites such as PubMed. The search terms included “ischemic-reperfusion injury,” “myocardial infarction,” “extracellular vesicles,” and “mesenchymal stem cell.” In addition, researchers manually reviewed reference lists to identify other relevant studies. This iterative process continued until no new relevant studies were identified. A total of 18 studies was identified in this section (11, 13, 34, 46–60).

### 3.1 The effect of BMSC-EVs on angiogenesis following myocardial ischemia- reperfusion injury

Bone Marrow Mesenchymal Stem Cells (BMSCs) are a type of stem cells derived from bone marrow, characterized by their remarkable potential for multilineage differentiation. Recent studies have demonstrated that BMSCs can exert proangiogenic effects and alleviate myocardial injury following ischemic insult. Bian et al. showed that BMSC-EVs can enhance the proliferation, migration, and tube formation capacity of human umbilical vein endothelial cells (HUVECs) *in vitro* (52). Further studies have revealed that BMSC-EVs can significantly promote the proliferation and tube formation capacity of HUVECs by secreting miRNAs. Ma et al. demonstrated that BMSC-EVs carrying miR-132 can enhance the angiogenic capacity of HUVECs *in vivo* (55). Their animal experiments further confirmed that BMSC-EVs carrying miR-132 significantly promote neovascularization in the peri-infarct region (55). Moreover, Wang et al. also found that BMSC-EVs carrying miR-210 can enhance the tube formation, migration, and proliferation of HUVECs, as well as improve angiogenesis and cardiac function after myocardial infarction by regulating their target gene Ephrin-A3 (EFNA3) (34). miRNAs derived from BMSC-EVs can promote angiogenesis and protect cardiomyocytes by acting on signaling factors and pathways. Zheng et al. revealed that miR-29b-3p secreted by BMSC-EVs upregulates the expression of A Disintegrin and Metalloproteinase with Thrombospondin type I motifs, 16 (ADAMTS16) (58). ADAMTS16, as a protease, can play a role in angiogenesis, tissue repair, and other aspects (58). Zheng et al. demonstrated that miR-29b-3p can promote vascular regeneration and myocardial repair in myocardial infarction models by upregulating the expression of ADAMTS16 (58). Similarly, Chen et al. showed that MSC-EVs carrying miR-126 can activate the AKT/ERK pathway (60). The AKT/ERK pathway not only promotes the survival, migration, and proliferation of endothelial cells, but also enhances angiogenesis and the repair of damaged tissues by upregulating the expression of key factors such as VEGF (60). In addition to exerting effects through the release of miRNAs, BMSCs-EVs can also influence other cells via direct cell-cell interactions. MSCs-EVs can enhance the survival and angiogenic capacity of cardiac stem cells (CSCs) following ischemic heart disease by secreting microRNAs (49). CSCs are a type of stem cells residing in the heart, capable of playing a significant role in cardiac tissue repair and vascular regeneration. Although existing studies have shown that BMSC-EVs can promote angiogenesis and tissue repair through various mechanisms following ischemic stroke, some research remains in the stages of *in vitro* experiments and animal models. The specific effects and therapeutic potential of BMSC-EVs in human cardiac ischemic injury still need to be further explored and validated.

### 3.2 The effect of hUC-MSC-EVs on angiogenesis following myocardial ischemia-reperfusion injury

Human Umbilical Cord Mesenchymal Stem Cells (hUC-MSCs), as a type of mesenchymal stem cells, possess multilineage differentiation potential and low immunogenicity, thus playing a significant role in promoting angiogenesis and reducing myocardial cell injury following ischemic insult. Gao et al. demonstrated in MI model that hUC-MSC-EVs carrying miR-423-5p can significantly inhibit the expression of EFNA3, a key negative regulator of angiogenesis and myocardial repair (13). By targeting and inhibiting EFNA3, miR-423-5p effectively promotes angiogenesis and myocardial repair, significantly reducing myocardial fibrosis (13). hUC-MSCs can promote the repair of damaged tissues and vascular regeneration by modulating intracellular signaling pathways. Yang et al. demonstrated through *in vitro* experiments using HUVECs and *in vivo* experiments using MI models that hUC-MSC-EVs-miR-223 exert reparative effects following myocardial ischemic injury (59). MiR-223 binds to its target gene p53, promoting its degradation and thereby alleviating the inhibition of S100A9 (61). The upregulation of S100A9 can enhance angiogenesis and myocardial repair. Therefore, hUC-MSC-EVs-miR-223 can promote angiogenesis and the repair and regeneration of damaged tissues via the p53/S100A9 axis (59). In addition to normal MSCs, hypoxia-induced MSCs can also exert proangiogenic and reparative effects. Shao et al. demonstrated that hypoxia preconditioned human umbilical cord mesenchymal stem cells (hUC-MSChyp) release EVs that promote the tube formation and migration of HUVECs by delivering miR-214, which inhibits the expression of Sufu and activates the Hedgehog signaling pathway (54). In summary, hUC-MSC-EVs promote myocardial repair and angiogenesis following ischemic injury by releasing miRNAs such as miR-423-5p, miR-223, and miR-214, which act on different signaling pathways and target genes. This provides new targets and strategies for the treatment of myocardial ischemic injury.

### 3.3 The effect of ADSCs-EVs on angiogenesis following myocardial ischemia- reperfusion injury

Adipose-Derived Mesenchymal Stem Cells (ADMSCs) also play a significant role in both myocardial ischemic injury and ischemia-reperfusion injury. Wang et al. demonstrated that the miRNA-205 released by ADSC-EVs can effectively promote the proliferation and migration of microvascular endothelial cells, thereby enhancing angiogenesis and inhibiting cardiomyocyte apoptosis (48). In addition, Zhu et al. discovered that miR-31 released by ADSC-EVs significantly promotes angiogenesis and tissue repair. Specifically, miR-31 in ADSC-EVs can target and inhibit its target gene, Factor Inhibiting HIF-1 (FIH1), which is a negative regulatory protein that suppresses the activity of hypoxia-inducible factor-1α (HIF-1α) (50). Under hypoxic conditions, the activity of HIF-1α is activated, which can significantly promote angiogenesis and the repair and regeneration of damaged tissues. Therefore, by targeting and inhibiting FIH1, miR-31 in ADSC-EVs activates the HIF-1/HIF-1α axis, thereby promoting angiogenesis and tissue repair (50). These findings provide new targets and strategies for the treatment of ischemic diseases.

### 3.4 The effect of other stem cell-EVs on angiogenesis following myocardial ischemia- reperfusion injury

**Figure 1:**
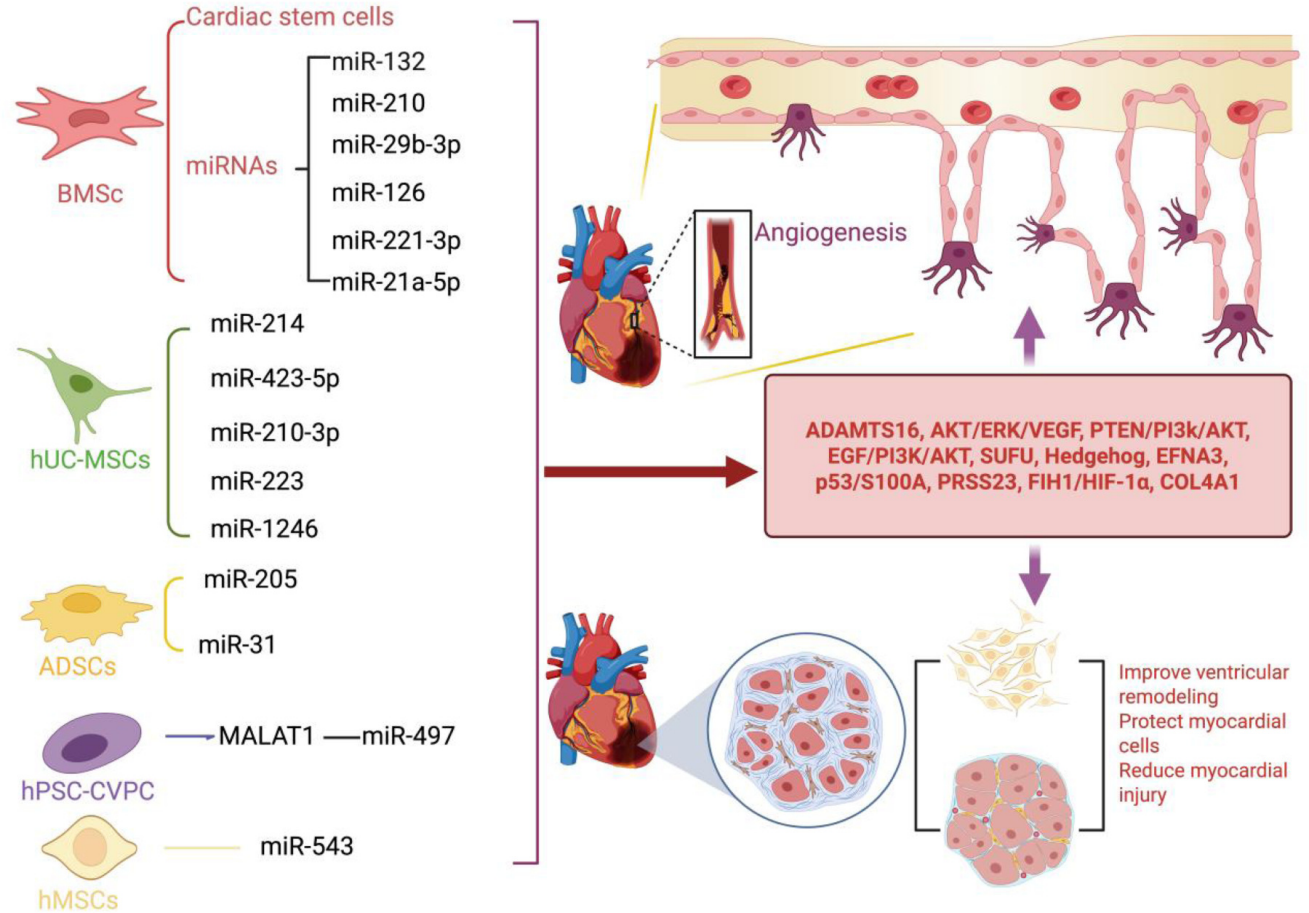
Following myocardial ischemic injury, MSCs-EVs can effectively promote cardiac angiogenesis, accelerate the repair process of cardiomyocytes, and provide robust protection for cardiomyocytes. (1) BMSCs-EVs not only act directly on cardiac stem cells but also release a variety of key microRNAs (including miR-132, miR-210, miR-29b-3p, miR-126, miR-221-3p, and miR-21a-5p). By precisely regulating important signaling pathways and effectors such as ADAMTS6, PTEN/PI3K/Akt, and VEGF, they significantly promote angiogenesis, protect cardiomyocytes from damage, and effectively prevent the occurrence of ventricular remodeling. (2) hUC-MSCs-EVs can facilitate angiogenesis and protect cardiomyocytes by releasing miRNAs such as miR-214, miR-423-5p, miR-210-3p, miR-223, and miR-1246. These miRNAs regulate signaling pathways and factors including EGF/PI3K/Akt, SUFU, EFNA3, and PRSS23. (3) ADSCs-EVs exert their cardioprotective and pro-angiogenic effects by releasing miR-205 and miR-31, which modulate the FIH1/HIF-1α and VEGF pathways. (4) hPSC-CVPCs enhance the release of miR-497 through the action of MALAT1, thereby protecting cardiomyocytes and stimulating angiogenesis. (5) hMSCs release miR-543 to downregulate COL4A1, thereby promoting angiogenesis. Created using BioRender.com.

Other types of MSCs also play significant roles in ischemic heart diseases. Human pluripotent stem cell (hPSC)-derived cardiovascular progenitor cells (CVPCs) offer a highly promising therapeutic strategy for ischemic heart diseases. Wu et al. discovered that extracellular vesicles (hCVPC-EVs) secreted by hPSC-CVPCs can upregulate the expression of the long noncoding RNA (lncRNA) MALAT1 (46). This lncRNA targets miR-497, significantly enhancing the survival of damaged cardiomyocytes and promoting the tube formation capacity of HUVECs in MI models (19, 46). Consequently, hCVPC-EVs demonstrate substantial therapeutic potential in alleviating myocardial ischemia and reperfusion injury by improving cardiomyocyte viability and promoting angiogenesis (46). Human mesenchymal stem cells (hMSCs) have also been shown to promote vascular regeneration. Yang et al. demonstrated that hMSC-EVs can significantly reduce infarct size and alleviate MI-induced damage in MI models. Moreover, hMSC-EVs can enhance the proliferation, migration, invasion, and angiogenesis of cardiac microvascular endothelial cells (CMECs) by releasing miR-543 (11). In summary, MSCs significantly promote angiogenesis, reduce myocardial fibrosis, protect cardiomyocytes, and improve cardiac function by releasing microRNAs and other factors following myocardial ischemic injury. These characteristics offer new strategies for the treatment of ischemic heart diseases, effectively protecting cardiac function by promoting neovascularization and alleviating myocardial damage. However, most current studies are based on cell experiments and animal models, and the therapeutic effects and mechanisms of action in humans still require further exploration. Future research needs to focus on the long-term effects, safety, and potential optimization strategies of MSCs in humans to advance their application in clinical therapy. For further insights into the research on MSCs-EVs following ischemic cardiac injury, please refer to Figure 1 and Table 1.

**Table 1:** The emerging role of MSC-EVS on angiogenesis following heart, kidney, and brain ischemia-reperfusion injury.

The effect of MSC-EVS on angiogenesis following myocardial ischemia-reperfusion injury					
Author	MSC-EVs Source	Recipient Cells	Cargos	Model	Effect on angiogenesis
Wu et al. (46)	hPSC-CVPC	Endothelial cells	MALAT1, miR-497	MI	Promote the infarct healing through improvement of cardiomyocyte survival and angiogenesis
Han et al. (47)	hUC-MSC	Endothelial cells	NA	MI	Activation of the Epidermal Growth Factor (EGF)/PI3K/Akt signaling pathway promotes angiogenesis
Wang et al. (48)	ADSC	Endothelial cells	miR-205	MI	Regulation of HIF-1α and VEGF promotes angiogenesis
Zhang et al. (49)	BMSCs	Endothelial cells	VEGF, HIF-1α, miR-221-3p	NA	Activation of the PI3 K/Akt and Wnt/β-catenin pathways promotes angiogenesis
Zhu et al. (50)	ADSCs	Endothelial cells	miR-31	MI	miR-31 increases capillary density via the FIH1/HIF-1α pathway
Luther et al. (51)	BMSCs	Endothelial cells	miR-21a-5p	MI	Activation of the PI3 K/Akt signaling pathway promotes angiogenesis
Bian et al. (52)	BMSCs	Endothelial cells	NA	MI	miR-21 enhances microvascular density via the PTEN/AKT signaling pathway
Sun et al. (53)	BMSCs	NA	miR-221-3p	MI	Promote angiogenesis by regulating the PTEN/PI3 K/Akt signaling pathway
Wang et al. (34)	BMSCs	Endothelial cells	miR-210	MI	Activation of the AKT signaling pathway, promotion of the VEGF signaling pathway, and enhancement of autophagy
Shao et al. (54)	hUC-MSCs	Endothelial cells	miR-214	MI	Inhibition of Sufu promotes angiogenesis
Ma et al. (55)	BMSCs	Endothelial cells	miR-132	MI	Promote angiogenesis by activating the PI3 K/Akt/eNOS pathway
Yang et al. (11)	hMSCs	Endothelial cells	miR-543	MI	Downregulation of COL4A1 promotes angiogenesis
Gao et al. (13)	hUC-MSCs	Endothelial cells	miR-423-5p	MI	Promote angiogenesis through the miR-423-5p/EFNA3 axis
Pu et al. (56)	hUC-MSCs	Endothelial cells	miR-210-3p	MI	Ameliorated Myocardial Infarction via miR-210-3p Promoted Angiogenesis
Wang et al. (57)	hUC-MSCs	Endothelial cells	miR-1246	MI	Promote angiogenesis and protect the heart from failure by targeting PRSS23
Zheng et al. (58)	BMSCs	Endothelial cells	miR-29b-3p	MI	Promote Angiogenesis and Ventricular Remodeling in Rats with Myocardial Infarction by Targeting ADAMTS16
Yang et al. (59)	hUC-MSCs	Endothelial cells	miR-223	MI	Relieve myocardial fibrosis and inflammation infiltration, and promoted the angiogenesis
Chen et al. (60)	BMSCs	Endothelial cells	miR-126	MI	Improve angiogenesis and cardiac function in the infarcted area due to stimulation of the AKT/ERK-related pathway
The effect of MSC-EVS on angiogenesis following renal ischemia-reperfusion injury					
Hu et al. (70)	BMSCs	Endothelial cells	HIF-1α, VEGF	AKI	Promote the regeneration and repair of capillaries
Xing et al. (10)	MSCs	Endothelial cells	NA	AKI	Promote the regeneration of capillaries
Zou et al. (71)	huMSCs	Endothelial cells	VEGF	AKI	Promote an increase in capillary density
Eirin et al. (35)	BMSCs	Endothelial cells	NA	MRVD	Promote an increase in capillary density
The effect of MSC-EVS on angiogenesis following brain ischemia-reperfusion injury					
Hu et al. (73)	BMSCs	Endothelial cells	miR-21-5p	OGD/R	Enhance the expression of VEGF and VEGFR2 via miR-21-5p
Feng et al. (74)	hUC-MSCs	Endothelial cells	miR-320	MCAO	Regulate the miR-320/KLF5 axis to promote angiogenesis
Lu et al. (75)	hiPSC-MSC	Endothelial cells	VEGF, CXCR4	MCAO	Promote angiogenesis by enhancing VEGF and CXCR4 proteins
Bao et al. (76)	BMSCs	Endothelial cells	miR-486	OGD/R	Promote the generation of vascular endothelial cells
Yang et al. (77)	BMSCs	Endothelial cells	miR-181b-5p	MCAO	Promote angiogenesis in ECs via miR-181b-5p/TRPM7 axis
Zhang et al. (36)	BMSCs	Endothelial cells	miR-210	MCAO	Inhibit EGLN1, thereby activate the HIF-1α signaling pathway
Pan et al. (78)	BMSCs	Endothelial cells	miR-126	OGD/R	inhibit SPRED1 and activate the Ras/ERK signaling pathway
Xu et al. (79)	Human serum	Endothelial cells	miR-340-5p	OGD/R, MCAO	Repress ECs proliferation, migration, and tube formation
Wang et al. (80)	EPCs	Endothelial cells	miR-126	OGD/R, MCAO	Increase microvessels density
Wang et al. (81)	EPCs	Endothelial cells	miR-126	OGD/R, MCAO	Increase microvessels density and decrease ECs apoptosis
Gregorius et al. (82)	hypoxic MSCs	Endothelial cells	miR-126-3p	MCAO	Increase ECs migration and tube formation
Gregorius et al. (82)	hypoxic BMSCs	Endothelial cells	miR-140-5p	MCAO	Increase ECs migration and tube formation
Gregorius et al. (82)	hypoxic BMSCs	Endothelial cells	miR-186-5p	MCAO	Increase ECs migration and tube formation
Gregorius et al. (82)	hypoxic BMSCs	Endothelial cells	miR-370-3p	MCAO	Increase ECs migration and tube formation
Gregorius et al. (82)	hypoxic BMSCs	Endothelial cells	miR-409-3p	MCAO	Increase ECs migration and tube formation

ADSC, adipose-derived stem cells; BMSCs, bone marrow-derived mesenchymal stem cells; ECs, endothelial cells; EPCs, endothelial progenitor cells; hUC-MSCs, human umbilical cord-derived mesenchymal stem cells; hiPSC-MSC, human induced pluripotent stem cell-derived mesenchymal stem cells; MCAO, middle cerebral artery occlusion; OGD, oxygen-glucose deprivation; OGD/R, oxygen-glucose deprivation/reperfusion; MI, myocardial infarction; AKI, acute kidney injury; CXCR4, C-X-C chemokine receptor type 4; FIH1, factor inhibiting HIF-1; HIF-1α, hypoxia-inducible factor 1-alpha; PI3K/Akt, phosphoinositide 3-kinase/protein kinase B; PTEN/AKT, phosphatase and tensin homolog/protein kinase B; Ras/ERK, Ras/extracellular signal-regulated kinase; SPRED1, sprouty-related EVH1 domain-containing protein 1; TRPM7, transient receptor potential cation channel subfamily M member 7; VEGF, vascular endothelial growth factor; Wnt/β, Wnt/β-catenin signaling pathway; ADAMTS16, A disintegrin and metalloproteinase with thrombospondin motifs 16; COL4A1, collagen type IV alpha 1 chain; EFNA3, Ephrin-A3; EGLN1, Egl-9 family hypoxia inducible factor 1; KLF5, kruppel-like factor 5; PRSS23, protease serine 23; NA, not applicable.

## 4 The effect of MSC-EVS on angiogenesis following renal ischemia-reperfusion injury

The kidneys, as vital excretory organs in the human body, filter waste and maintain water and electrolyte balance, filter waste and maintain water and electrolyte balance (62). However, the high incidence of kidney diseases has become a significant factor affecting human health. Kidney diseases can be broadly categorized into primary and secondary kidney diseases based on their causes (63–65). Among these, ischemia and insufficient perfusion of the kidneys, caused by various factors, are important mechanisms underlying the development of kidney diseases. Recent in-depth research on MSC-EVs has revealed that MSC-EVs can promote angiogenesis in damaged kidneys through the secretion of active factors, thereby reducing ischemia and hypoxia and improving renal function (21, 66, 67). In recent years, research results have shown that mesenchymal stem cells from different sources can promote the generation and repair of renal capillaries, improve ischemia and hypoxia in damaged tissues and cells, and facilitate the repair and regeneration of damaged tissues, thereby enhancing kidney function (68, 69).

Xing et al. found that MSC-EVs can promote angiogenesis and repair in the kidneys following ischemia and hypoxia by secreting a variety of cytokines, thereby improving renal microcirculation (10). Subsequently, Hu et al. injected BMSC-EVs into a rat model of acute kidney injury induced by cisplatin and conducted observations. Compared with the control group, rats treated with BMSC-EVs exhibited reduced levels of serum creatinine (Scr) and blood urea nitrogen (BUN), indicating improved renal function (70). Further experimental revealed increased expression of peritubular capillary markers (such as CD31 and VE-cadherin), indicating enhanced angiogenesis and repair of blood vessels in the treated rats. Immunohistochemistry and fluorescence imaging demonstrated a significant increase in the density of peritubular capillaries in the kidneys of rats treated with BMSC-EVs (70). Further exploration showed that BM-MSCs promote the generation and repair of peritubular capillaries through the secretion of factors such as HIF-1α and VEGF, thereby improving ischemia and hypoxia in the damaged kidneys (70). Similarly, Eirin et al. demonstrated that injecting BMSC-EVs into the MRVD model significantly increased renal vascular density and the expression of vascular markers in the treated kidneys, suggesting that BMSC-EVs have the potential to promote the regeneration and repair of renal microvasculature following ischemia and hypoxia (35). In addition to BMSC-EVs, hUC-MSCs can also exert protective effects on renal ischemia-reperfusion injury and promote angiogenesis. Zou et al. injected hUC-MSC-EVs into a rat renal ischemia-reperfusion model. The results showed significant improvement in renal function indicators (Scr and BUN) and a marked increase in vascular markers (71). The study demonstrated that hUC-MSC-EVs protect renal function by promoting renal microvascular generation and repair, improving renal microcirculation, and reducing ischemia and hypoxia (71).

Although research on MSC-EVs has become a hot topic and continues to deepen, studies on their role in promoting angiogenesis following renal ischemic injury are relatively limited. Compared to other fields, the specific mechanisms of action, active factors, and related signaling pathways of MSC-EVs in this context still require further investigation. As research in this direction gradually progresses, MSC-EVs may pave a new way for the treatment of kidney diseases. (***For detailed information on the research regarding MSCs-EVs following ischemic kidney injury, please refer to*** Table 1).

## 5 The effect of MSC-EVS on angiogenesis following cerebral ischemia-reperfusion injury

Ischemic stroke, characterized by its high disability and mortality rates, has become one of the major diseases affecting human health. Although significant progress has been made in ischemia-reperfusion therapies, such as thrombectomy and thrombolysis, their clinical application is limited by the narrow therapeutic time window and potential risk of bleeding (72). In recent years, with the in-depth research on MSC-EVs, their therapeutic and reparative effects on the nervous system following ischemic stroke have gradually been uncovered. This article reviews the current research findings on the role of MSC-EVs in promoting angiogenesis and repairing damaged blood vessels after ischemic stroke, providing insights and directions for further in-depth studies. MSCs, which possess the potential for multilineage differentiation, are found in a variety of tissues, including adipose tissue, bone marrow, serum, human umbilical cord, human induced pluripotent stem cells, and placenta. This review aims to summarize the effects of EVs derived from different types and sources of MSCs on angiogenesis following ischemic stroke. To systematically evaluate the therapeutic potential of MSC-EVs in ischemic stroke, a rigorous literature retrieval protocol was implemented following PRISMA guidelines. The search strategy employed a combination of controlled vocabulary (MeSH terms) and keywords, including “angiogenesis”, “ischemic stroke”, “extracellular vesicles”, and “mesenchymal stem cells”, across multiple databases (PubMed, Web of Science, Embase). To ensure comprehensiveness, backward citation tracking of included studies and relevant reviews was performed. Iterative search refinement continued until theoretical saturation was achieved, yielding 11 eligible studies meeting predefined PICOS criteria (36, 73–82).

### 5.1 The effects of BMSC-EVs on angiogenesis following ischemic stroke

BMSCs, multipotent stromal cells isolated from bone marrow aspirates through density gradient centrifugation (e.g., Ficoll-Paque®), are phenotypically characterized by their adherence to International Society for Cellular Therapy (ISCT) criteria (83, 84). When cultured in α-MEM supplemented with 10% FBS under normoxic conditions, BMSCs exert therapeutic effects through the secretion of trophic factors (BDNF, GDNF) and the transfer of exosomal miRNAs (e.g., miR-126), which modulate the PI3K-Akt and Wnt/β-catenin pathways to enhance neurovascular repair (83, 84). In preclinical ischemic stroke models, BMSC transplantation reduced infarct volume by 40% and improved neurobehavioral scores by 35% (modified Neurological Severity Score, *p* < 0.01) (85, 86). Over 50 registered clinical trials (e.g., NCT04519671, NCT03069170) are evaluating BMSC-based therapies for conditions ranging from ischemic stroke to myocardial ischemia- reperfusion injury and renal ischemia-reperfusion injury, with Phase II trials demonstrating a 25% improvement in Barthel Index scores at 90-day follow-up.

BMSC-EVs can promote angiogenesis following ischemic stroke through the secretion of various miRNAs. Hu et al. investigated the role of BMSC-EVs-miR-21-5p in angiogenesis by injecting them into the Middle Cerebral Artery Occlusion (MCAO) model (73). Further research indicated that miR-21-5p can inhibit phosphatase and tensin homolog (PTEN), thereby activating the downstream PI3 K/AKT signaling pathway, which in turn promotes angiogenesis and vascular repair following ischemic stroke (73). Similarly, the study by Bao et al. demonstrated that BMSC-EVs-miR-486 can also activate the PI3 K/Akt signaling pathway by inhibiting PTEN, thereby promoting angiogenesis following ischemic stroke (76). In addition to the PTEN/PI3 K/AKT signaling pathway, BMSC-EVs can promote angiogenesis by secreting miR-210, which targets and inhibits Egl-9 Family Hypoxia Inducible Factor 1 (EGLN1) to activate the HIF-1α signaling pathway, thereby promoting the formation of new blood vessels (36). It has been reported that miR-181b-5p (77) and miR-126 (78), both derived from BMSC-EVs, contribute to the promotion of angiogenesis through their interactions with endothelial cells. Specifically, miR-181b-5p enhances angiogenesis in Brain Microvascular Endothelial Cells (BMECs) by targeting the Transient Receptor Potential Cation Channel Subfamily M Member 7 (TRPM7) pathway (77), while miR-126 facilitates angiogenesis by inhibiting Sprouty-related EVH1 domain-containing protein 1 (SPRED1) and subsequently activating the Ras/ERK signaling pathway (78).

In addition to conventionally cultured and isolated BMSC-EVs, those derived under hypoxic conditions also play a significant role following ischemic stroke. Gregorius et al. demonstrated that miRNAs such as miR-126-3p, miR-140-5p, miR-186-5p, miR-370-3p, and miR-409-3p, which are enriched in hypoxic BMSC-EVs, can inhibit EGLN1 and PTEN to activate the HIF-1α or PI3K/Akt signaling pathways (82). These pathways enhance the migration and proliferation of endothelial cells, thereby promoting angiogenesis (82). Therefore, MSC-EVs promote endothelial cell migration and regeneration by secreting a variety of distinct miRNAs that activate intracellular signaling pathways. This mechanism contributes to angiogenesis and vascular repair following ischemic stroke.

### 5.2 The effects of hUC-MSC-EVs on angiogenesis following ischemic stroke

hUC-MSCs, derived from Wharton's jelly or umbilical cord tissue, strike an optimal balance between accessibility and therapeutic (87). As a byproduct of childbirth, their collection is non-invasive and ethically uncontroversial. Compared to BM-MSCs, hUC-MSCs boast faster proliferation rates and lower immunogenicity, attributed to minimal Human Leukocyte Antigen (HLA) class II expression, rendering them ideal for allogeneic applications without stringent HLA matching (87, 88).

Despite these advantages, variability in isolation methods, such as enzymatic digestion vs. explant culture, can introduce batch-to-batch heterogeneity. Moreover, their chondrogenic differentiation potential is generally inferior to that of BM-MSCs. Nevertheless, hUC-MSCs have demonstrated superior therapeutic effects in modulating inflammation and promoting tissue regeneration, primarily through the secretion of exosomal microRNAs (e.g., miR-146a, miR-181c) and trophic factors such as VEGF and Hepatocyte Growth Factor (HGF) (88–90).

To date, over 120 clinical trials (e.g., NCT04333368 for COVID-19-related ARDS; NCT04519671 for cerebral palsy) have confirmed the safety and regenerative potential of hUC-MSCs. Notably, Phase II studies have reported a 40% improvement in motor function scores in spinal cord injury patients at 6-month follow-up. Their non-invasive sourcing, ethical viability, and robust cryopreservation stability position hUC-MSCs as a leading candidate for both allogeneic cell therapy and exosome-based regenerative medicine.

Emerging evidence also highlights the therapeutic promise of hUC-MSC-EVs in treating cerebral ischemia-reperfusion injury (90). In a hypoxia/reoxygenation model, human brain microvascular endothelial cells co-cultured with hUC-MSC-EVs exhibited increased expression of circDLG Associated Protein 4 (circDLGAP4) and Kruppel-like factor 5 (KLF5), alongside decreased levels of miR-320 (74). These effects were mediated by the transfer of exosomal circDLGAP4. Silencing circDLGAP4 in hUC-MSC-EVs abolished their ability to enhance cell viability, migration, and tube formation in hypoxia/reoxygenation-treated human brain microvascular endothelial cells *in vitro*, and negated their protective effects against cerebrovascular injury in ischemia-reperfusion rat models (74). These findings suggest that hUC-MSC-EVs exert cerebrovascular protection, at least in part, through the circDLGAP4/miR-320/KLF5 axis (74).

### 5.3 The effects of hiPSC-MSC derived EVs on angiogenesis following ischemic stroke

Human Induced Pluripotent Stem Cell-derived MSCs **(**hiPSC-MSCs), generated through the differentiation of induced pluripotent stem cells, offer unparalleled scalability and standardization. Unlike primary MSCs, which are subject to donor variability, hiPSC-MSCs can be produced in virtually unlimited quantities from a single cell line, making them ideal for large-scale therapeutic manufacturing. Additionally, their origin from reprogrammed somatic cells allows for genetic engineering, such as Clustered Regularly Interspaced Short Palindromic Repeats (CRISPR)-based modifications to enhance homing efficiency or immunomodulatory capacity. However, this approach carries inherent risks, including potential residual pluripotency that might lead to teratoma formation, and the complex protocols required for differentiation demand rigorous quality control. In contrast, BM-MSCs, harvested from bone marrow aspirates, remain the gold standard due to decades of research validating their osteogenic differentiation potential and clinical efficacy in conditions like graft-vs.-host disease and osteoarthritis. Their drawbacks include invasive extraction procedures, limited cell yields, and age-related functional decline, as cells from elderly donors often exhibit reduced proliferative capacity.

Lu et al. (75) investigated the neuroprotective and pro-angiogenic effects of extracellular vesicles derived from hiPS-MSC-EVs using both *in vitro* and *in vivo* models of ischemic stroke. hiPS-MSC-EVs were isolated via ultrafiltration and applied to HT22 neuronal cells subjected to 2 h of oxygen-glucose deprivation followed by reoxygenation (OGD/R) (75). The treatment significantly enhanced cell viability, reduced apoptotic cell death, and mitigated OGD/R-induced morphological damage (75).

In parallel, a murine model of ischemic stroke was established by inducing MCAO in male C57BL/6 mice (75). hiPS-MSC-EVs were administered intravenously at three defined time points post-occlusion. The treatment resulted in a notable reduction in infarct volume, improved motor function recovery, and increased angiogenic activity in the ischemic hemisphere, as evidenced by elevated expression of VEGF and C-X-C motif chemokine receptor 4 (CXCR4) (75). These findings collectively demonstrate that hiPS-MSC-EVs exert significant neuroprotective effects and promote post-ischemic angiogenesis, highlighting their therapeutic potential for ischemic stroke intervention (75).

### 5.4 The effects of other types of cell-derived EVs on angiogenesis following ischemic stroke

Other types of MSC-EVs also play a role in angiogenesis following ischemic stroke. Xu et al. revealed that serum-derived EVs are rich in miR-340-5p (79). Further experiments demonstrated that miR-340-5p can enhance the repair and regenerative capacity of BMECs, thereby promoting angiogenesis following ischemic stroke (79). However, the detailed genetic targets and signaling pathways involved still require further investigation. Endothelial progenitor cell-derived extracellular vesicles (EPC-EVs) also participate in repair and angiogenesis following injury. EPC-EVs-miR-126 can inhibit SPRED1 to activate the Ras/ERK signaling pathway, thereby promoting angiogenesis and neuronal repair after injury (80).

By searching databases and reviewing relevant literature, the above-mentioned roles of MSC-EVs in angiogenesis following ischemic stroke have been summarized. In summary, MSC-EVs of different sources and types activate signaling pathways such as Ras/ERK, KLF5, HIF-1α, and PI3 K/Akt through various targets via the miRNAs and other factors they carry, participating in angiogenesis and the repair of damaged blood vessels. As research on mesenchymal stem cells deepens, more types of MSCs and their roles, as well as signaling pathways, will be discovered. This review, by summarizing existing research findings, attempts to provide ideas and directions for further research and clinical applications.

## 6 EV-associated cargos involved in at least two of three ischemia-reperfusion conditions demonstrate significant overlaps in their modes of action

Ischemia-reperfusion injury, while presenting distinct pathophysiological characteristics across different organ systems, reveals a conserved molecular mechanism mediated by extracellular vesicle (EV) cargoes. Our analysis identified 35 EV-associated molecules that participate in multiple ischemia-reperfusion conditions, including 11 ncRNAs and 2 proteins in cerebral ischemia, 16 ncRNAs and 2 proteins in myocardial infarction, and 2 proteins in renal ischemia-reperfusion injury. These overlapping cargoes demonstrate consistent angiogenic effects through tissue-specific pathway modulation, suggesting their fundamental role in ischemic protection. The significant molecular overlap across these conditions not only provides novel insights into shared pathological mechanisms, but also highlights promising therapeutic targets for developing broad-spectrum treatments against ischemia-reperfusion injury. Our analysis identified a conserved molecular signature comprising three miRNAs [miR-21 (51, 73), miR-126 (60, 78, 80–82), miR-210 (34, 36, 56)] and two proteins [HIF-1α (49, 70), VEGF (49, 70, 71, 75)] that are functionally involved in multiple pathological conditions. Specifically, these molecular mediators demonstrate significant overlap between ischemic stroke and myocardial infarction, as well as between renal ischemia-reperfusion injury and myocardial infarction. The consistent involvement of these miRNAs and proteins across distinct ischemic conditions suggests their fundamental role in the underlying pathophysiology of ischemia-reperfusion injury.

**Figure 2:**
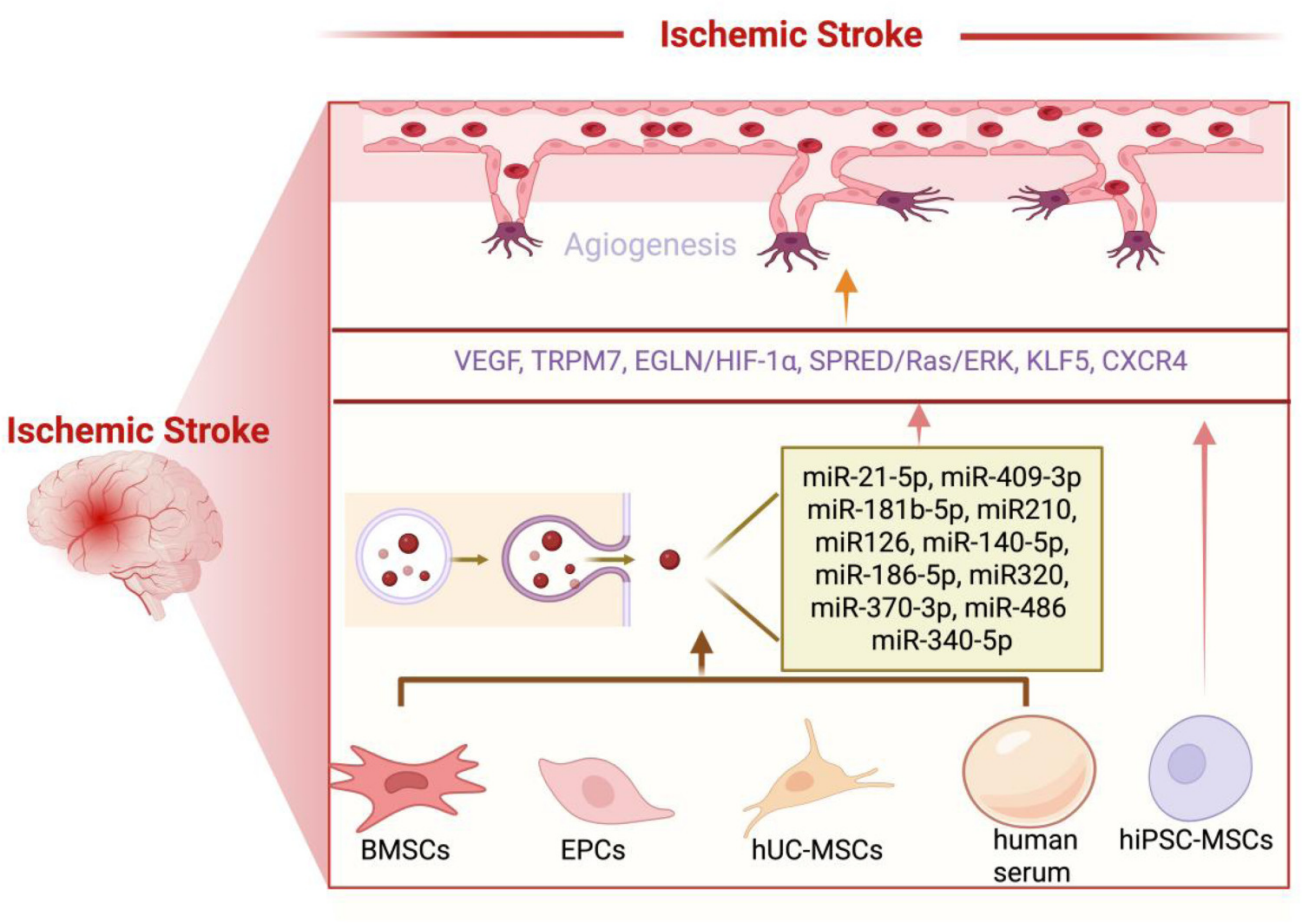
Following ischemic stroke, MSCs-EVs can effectively promote angiogenesis, contributing to the recovery of cerebral vasculature and overall brain function. (1) BMSCs-EVs promote angiogenesis by releasing a variety of microRNAs, including miR-21-5p, miR-486, miR-181b-5p, miR-210, miR-126, miR-140-5p, miR-186-5p, miR-370-5p, and miR-409-3p. These microRNAs act on key targets such as VEGF, TRPM7, EGLN1/HIF-1α, and the SPRED1/Ras/ERK pathway, thereby stimulating the regeneration of blood vessels. (2) hUC-MSCs-EVs promote angiogenesis by releasing miR-320, which acts on KLF5. (3) hiPSC-MSC-EVs promote angiogenesis by releasing VEGF and CXCR4. (4) Human serum promotes angiogenesis by releasing miR-340-5p. (5) EPCs promote angiogenesis by releasing miR-126. Created using BioRender.com.

Ischemia-reperfusion injury, characterized by initial blood flow interruption followed by subsequent restoration, is a common pathological mechanism underlying various organ-specific conditions, including cerebral stroke, myocardial infarction, and renal failure (4, 91). While these disorders manifest distinct clinical presentations, recent studies have revealed shared molecular pathways mediated by EVs and miRNA cargoes, in ischemia-reperfusion injury pathogenesis (4, 91). Among these mediators, three conserved miRNAs, namely miR-21 (51, 73), miR-126 (60, 78, 80–82), and miR-210 (34, 36, 56), have emerged as key regulators of post-ischemic angiogenesis through coordinated yet distinct mechanisms: miR-21-5p-mediated endothelial cytoprotection and motility enhancement (51, 73), miR-126-dependent vascular stabilization and neovascularization promotion (60, 78, 80–82), and miR-210-regulated HIF-1α stabilization in hypoxic microenvironments (34, 36, 56). This synergistic regulation facilitates robust vascular network reconstruction, tissue reperfusion, and functional recovery following ischemic insults. Notably, EVs encapsulation provides critical protection for these miRNAs against extracellular degradation while enabling targeted delivery to recipient cells (91). The selective packaging of specific miRNA subsets into exosomes suggests a sophisticated intercellular communication system that precisely modulates ischemic stroke progression and recovery processes (91). These findings highlight exosomal miRNAs as both fundamental mediators of cross-organ protective mechanisms and potential therapeutic targets for ischemia-reperfusion injury management. For detailed information on the research of MSCs-EVs following ischemic stroke, please refer to Figure 2 and Table 1.

### 6.1 MSC-derived EV-associated miR-126 plays a role in at least two of three ischemia-reperfusion conditions

Previous study demonstrates that miR-126, a critical regulator of endothelial cell angiogenesis and vascular integrity (92), can be effectively delivered from MSCs to injured endothelial cells via EVs. In stroke models, Pan et al. (78) generated miR-126-overexpressing (EVs-miR-126) and miR-126-knockdown (EVs-SimiR-126) EVs by transfecting MSCs with miR-126 mimics or inhibitors, respectively. Functional analysis revealed that EVs-miR-126 significantly enhanced miR-126 levels in hypoxia/reoxygenation-injured endothelial cells compared to control EVs, while EVs-SimiR-126 showed attenuated effects in elevating miR-126 expression. These findings provide direct evidence that MSC-derived exosomes serve as efficient vehicles for intercellular miR-126 transfer, highlighting their potential as a targeted therapeutic strategy for promoting vascular repair in ischemic conditions. Furthermore, Wang et al. elucidated the mechanistic role of EV-encapsulated miR-126 in the treatment of diabetic ischemic stroke (80). Their study demonstrated that miR-126 overexpression significantly potentiated the therapeutic efficacy of EVs by concurrently enhancing angiogenesis and neurogenesis, ultimately leading to improved neurological functional recovery during the chronic phase of diabetic ischemic stroke. Moderate-intensity treadmill training prior to ischemic stroke onset confers dual-phase neuroprotective benefits, as evidenced by reduced acute-phase neuronal apoptosis and enhanced chronic-phase functional recovery through coordinated promotion of angiogenesis and neurogenesis (81). Mechanistically, these therapeutic effects exhibit strong correlation with circulating EVs and their enriched miR-126 cargo, suggesting an EV-mediated exercise preconditioning effect. These findings highlight the critical involvement of EV-mediated miR-126 transfer in promoting neurovascular repair under hyperglycemic conditions.

Chen et al. (60) demonstrated that MSCs could be efficiently transfected with lentiviral vectors carrying miR-126 without compromising cell viability, and upon transplantation into a mouse model of acute myocardial infarction, these modified cells survived long-term and sustained miR-126 expression for at least six weeks at the injection site. Their findings revealed that intra-myocardial delivery of miR-126-overexpressing MSCs significantly enhanced vessel density in ischemic tissue after one month compared to untreated MSCs, indicating a robust pro-angiogenic and arteriogenic effect. This suggests that miR-126 plays a critical role in promoting vascular repair, likely through mechanisms such as enhancing VEGF signaling, improving endothelial cell function, and boosting the paracrine activity of MSCs. The study highlights the therapeutic potential of genetically engineered MSCs overexpressing miR-126 for ischemic heart disease, offering a promising strategy to improve cardiac repair by stimulating neovascularization in damaged myocardium. Further research is needed to assess long-term safety, functional recovery, and potential synergies with other pro-regenerative factors.

### 6.2 MSC-derived EV-associated miR-210 plays a role in at least two of three ischemia-reperfusion conditions

The induction of miR-210 is a nearly universal characteristic of the hypoxic response. Supporting this observation, miR-210 expression levels in brain and heart tissues demonstrate a direct correlation with other hypoxia-regulated genes, indicating its potential utility as an *in vivo* biomarker for ischemic conditions (56, 93). A comprehensive investigation of hypoxia-mediated miR-210 regulation in endothelial cells was conducted by Voellenkle et al. (94). Through deep-sequencing analysis, which enables precise and extensive profiling of the complete miRNA repertoire in hypoxic endothelial cells, miR-210 emerged as the most prominently upregulated miRNA. Notably, under the same experimental conditions, subsequent transcriptomic analysis of long noncoding RNAs revealed that miR210 Host Gene, the precursor transcript of miR-210, exhibited the most pronounced and statistically significant hypoxia-induced modulation (95). Growing evidence indicates that miR-210 overexpression enhances focal angiogenesis and promotes functional recovery in various ischemia/reperfusion models, such as middle cerebral artery occlusion (96), myocardial infarction (97), and renal ischemia (98). Studies have demonstrated that lentiviral vector-mediated delivery of miR-210 can cross the skull and reach the ischemic brain, stimulating localized angiogenesis and improving neurobehavioral outcomes in mice (99, 100). However, intravenous administration remains unsuitable for clinical translation due to delivery challenges. Therefore, developing a safe and efficient delivery system capable of crossing the blood-brain barrier is essential for therapeutic applications (101, 102). Interestingly, it is demonstrated that miR-210-loaded EVs could deliver miR-210 to the ischemic tissue through intravenous injection and induce focal angiogenesis.

Under ischemic stroke conditions, Zhang et al. (36) engineered c(RGDyK) peptide-conjugated exosomes derived from mesenchymal stromal cells and loaded them with cholesterol-modified miR-210. To model ischemic stroke, mice underwent middle cerebral artery occlusion. Following intravenous administration, near-infrared fluorescence imaging confirmed the ability of miR-210 loaded EVs to selectively target the ischemic brain. Elevated levels of miR-210 and VEGF in the lesion area confirmed successful delivery and functional activity. When administered every other day for 14 days, miR-210 loaded EVs significantly upregulated integrin β3, VEGF, and CD34, demonstrating enhanced angiogenesis. Meanwhile, Wang et al. (34) explored the therapeutic potential of MSC-derived EVs in promoting angiogenesis and improving cardiac function using a mouse myocardial infarction model. Their study revealed that miR-210 was highly enriched in MSC-EVs, and EVs derived from miR-210-silenced MSCs significantly lost their pro-angiogenic capacity both *in vitro* and *in vivo*. Further analysis demonstrated that MSC-EV treatment downregulated Efna3, a key miR-210 target gene involved in angiogenesis regulation, in human umbilical vein endothelial cells. These findings suggest that MSC-EVs effectively enhance angiogenesis and confer therapeutic benefits in myocardial infarction, likely through a miR-210/Efna3-dependent mechanism.

### 6.3 MSC-derived EV-associated miR-21-5p plays a role in at least two of three ischemia-reperfusion conditions

Emerging evidence indicates that miR-21 is significantly upregulated in ischemia-reperfusion injured tissues, where it promotes angiogenesis by enhancing key proangiogenic factors, including vascular endothelial growth factor, HIF-1α, and angiopoietin-1 (51, 73, 103). Notably, miRNA profiling studies have identified miR-21-5p as one of the most abundant miRNAs in EVs. To investigate its functional role, Hu et al. (73) examined whether MSC-derived EVs enhance post-ischemic angiogenesis via miR-21-5p upregulation. *in vitro* functional assays using human umbilical vein endothelial cells confirmed that MSC-EVs significantly enhanced cellular proliferation, migration, and tube formation capacity. Notably, when miR-21-5p was inhibited in MSCs using Lipofectamine 2,000 transfection, the exosomes showed diminished ability to upregulate key angiogenic factors including VEGF/VEGFR2 and Ang-1/Tie-2, resulting in attenuated proangiogenic effects on endothelial cells. These findings provide compelling evidence that MSC-EVs promote post-stroke angiogenesis primarily through miR-21-5p-mediated regulation of critical vascular signaling pathways, highlighting their therapeutic potential for ischemic stroke treatment. Both preclinical and clinical studies have demonstrated the therapeutic potential of MSCs for myocardial infarction. Kristin et al. (51) performed comprehensive deep sequencing of MSC-EV cargo and identified miR-21a-5p as the most abundant among several cardioprotective miRNAs. Their research revealed that MSC-EVs mediate cardioprotection and angiogenesis by transferring miR-21a-5p to recipient cardiac cells, subsequently downregulating key pro-apoptotic factors (PDCD4, PTEN, Peli1, FasL) in the myocardium. Through gain-of-function (miR-21 mimic transfection) and loss-of-function (miR-21a knockout MSC-EVs) experiments, the authors conclusively demonstrated that exosomal miR-21a-5p is effectively delivered to myocardial tissue and serves as a critical paracrine protective factor. These findings collectively demonstrate that MSC-EVs exert dual therapeutic effects through miR-21 delivery, promoting angiogenesis in ischemic stroke and myocardial infarction.

### 6.4 MSC-derived EVs plays a role in at least two of three ischemia-reperfusion conditions *via* VEGF and HIF-1α

VEGF is a central proangiogenic molecule that orchestrates blood vessel regeneration by activating receptors on vascular endothelial cells. Under ischemia-reperfusion injury, microRNAs-many of which are downregulated in MSC-EV-treated conditions-likely lose their inhibitory control over VEGF signaling. This suppression of regulatory miRNAs may lead to enhanced VEGF pathway activation, promoting therapeutic angiogenesis and vascular repair. For example, in a rat myocardial infarction model, MSC exosome-preconditioned cardiac stem cells had significantly better enhanced capillary density via VEGF (49). MicroRNA profiling revealed significant changes in a set of microRNAs in cardiac stem cells after MSC exosome treatment (49). Likewise, quantitative RT-PCR analysis revealed a marked reduction in VEGF164 mRNA expression within the renal ischemia-reperfusion group (70). Notably, bone marrow-derived MSC transplantation effectively restored VEGF164 transcript levels, demonstrating a time-dependent elevation pattern correlated with post-transplantation duration (70). Additionally, studies have shown that human-induced pluripotent stem cell-derived MSC-EV treatment reduces infarct volume, enhances spontaneous motor function, and promotes angiogenesis by upregulating VEGF and CXCR4 protein expression in the infarcted hemisphere of mice subjected to MCAO (75). In ischemia-reperfusion injury, HIF-1α and VEGF play a dual role in angiogenesis-while their activation during ischemia promotes compensatory blood vessel formation to restore tissue perfusion (104), their excessive signaling during reperfusion exacerbates vascular leakage, inflammation, and oxidative damage (104). HIF-1α stabilization under hypoxia upregulates VEGF, driving endothelial survival and angiogenesis, but sudden oxygen reintroduction disrupts this pathway, contributing to microvascular dysfunction. Therapeutic strategies must carefully balance HIF-1α/VEGF activity to enhance revascularization without worsening reperfusion injury, potentially through preconditioning or targeted drug delivery (104).

## 7 Bottlenecks and challenges in extracellular vesicle research

In recent years, the therapeutic potential of EVs has been continuously uncovered across a spectrum of diseases. As research advances, EVs have progressed from concept studies to rigorous pre-clinical trials, generating preliminary evidence for their safety, dose-dependency, and targeted delivery (105). Thus, EVs promise to become a high-precision, high-efficiency therapeutic platform for diverse diseases (106). Although rodent and primate models have confirmed the therapeutic potential of EVs in a variety of diseases, the translation of EVs from the laboratory to the clinic is still fraught with challenges and is confronted with numerous pressing bottlenecks that need to be resolved: (1) isolation techniques—such as ultracentrifugation, ultrafiltration, and size-exclusion chromatography—differ markedly in purity, yield, and subpopulation enrichment, leading to poor inter-laboratory reproducibility; (2) the absence of specific markers hampers precise discrimination of functional EV subtypes, obscuring the discrete effects of individual classes; (3) the concentration and dosage of functional cargoes (RNAs, proteins, etc.) vary substantially between batches and subpopulations, complicating standardization and dose control (105–107). Although EVs have demonstrated therapeutic efficacy across diverse disease models, their precise pharmacological mechanisms remain elusive. To date, no study has unequivocally identified the bioactive EVs subpopulation, their key effector molecules, or the downstream signaling pathways that drive the observed benefits (107). Critical data are also lacking on the optimal route of administration, *in vivo* distribution, pharmacokinetics, and immunogenicity of xenogeneic EVs (108). These substantial mechanistic gaps continue to hinder the clinical translation of EV-based therapies. Clinical translation of EVs remains fraught with challenges. First, there is no unified protocol for EVs production or for the isolation of their therapeutic cargoes, resulting in large batch-to-batch variations in purity and quality (109). Second, efficacy data are contradictory: identical EVs directed at the same indication yield dramatically different outcomes across studies and in clinical practice (110). Third, EVs products lack harmonized standards for preparation, classification, quality control, regulation, and safety. Fourth, most studies fail to quantify the exact concentration of active cargo within EVs, leaving a persistent gap between experimental findings and clinical dosing. In summary, EVs' cross-organ reparative potential is proven, yet clinical translation demands systematic breakthroughs in process standardization, mechanism clarity, regulatory harmonization and long-term safety (109).

## 8 Conclusion

This review elucidates the pivotal role of EV-mediated molecular mechanisms in ischemia-reperfusion injury across multiple organ systems, revealing a conserved network of miRNAs (miR-21, miR-126, miR-210) and proteins (HIF-1α, VEGF) that coordinately regulate angiogenesis and tissue repair. MSC-derived EVs emerge as critical therapeutic carriers, delivering these molecular cargoes to enhance vascular reconstruction and functional recovery in cerebral, cardiac, and renal ischemic models. By modulating VEGF signaling and stabilizing HIF-1α under hypoxic conditions, EV-associated molecules establish cross-organ protective effects, highlighting their dual capacity to mitigate ischemic damage and promote regeneration. These findings not only decode shared pathological pathways but also lay a foundation for developing EV-based broad-spectrum therapies. Future research should focus on optimizing EV engineering for targeted delivery and fine-tuning the spatiotemporal activation of HIF-1α/VEGF pathways to maximize therapeutic efficacy while minimizing reperfusion-related complications.
